# Is the Rotavirus Vaccine Really Associated with a Decreased Risk of Developing Celiac and Other Autoimmune Diseases?

**DOI:** 10.5041/RMMJ.10450

**Published:** 2021-10-25

**Authors:** Ibrahim Kiliccalan

**Affiliations:** Gulhane Faculty of Medicine, University of Health Sciences, Ankara, Turkey

**Keywords:** Celiac disease, mucosal immunity, rotavirus, rotavirus vaccine, vaccination

## Abstract

This review examines the risk of developing celiac disease (CD) and other autoimmune diseases in individuals receiving the rotavirus (RV) vaccine compared to the normal population. Celiac disease is a malabsorptive, chronic, immune-mediated enteropathy involving the small intestine. The pathogenesis of CD is multifactorial, and mucosal immunity plays an important role in its development. Low mucosal IgA levels significantly increase the risk of developing the disease. Rotavirus is an infectious agent that causes diarrhea, particularly in children aged 0–24 months, and is frequently involved in diarrhea-related deaths in these children. An oral vaccine against RV has been developed. While it is effective on RV infection, it also contributes to increasing mucosal immunity. Studies have indicated that individuals immunized with the RV vaccine are at lower risk of developing CD than unvaccinated individuals. In addition, the mean age for developing CD autoimmunity may be higher in the vaccinated group than in controls receiving placebo. Additional studies that include children immunized with different RV vaccines and unvaccinated children would provide more meaningful results. Although current data suggest a possible association of RV vaccination with a reduced risk of developing CD and other autoimmune diseases, this remains an unanswered question that merits greater international investigation.

## INTRODUCTION

Celiac disease (CD) is a chronic immune-mediated enteropathy of the small intestine driven by gluten ingestion in genetically predisposed individuals.^1^ It affects approximately 0.7% of the world’s population.^2^ The disease presents in a spectrum ranging from asymptomatic to symptomatic.^1^,^2^ Diagnosis is determined through histopathological demonstration of small intestinal villous atrophy in the presence of celiac autoantibodies (achieved by duodenal biopsy) and/or full symptom relief in response to a gluten-free diet.^2^ However, recent guidelines suggest that duodenal biopsies can be avoided in the presence of serological markers with classic symptoms of CD.^2^ Treatment of CD includes placing patients on a lifelong gluten-free diet.

Rotavirus (RV) is a double-stranded RNA virus from the Reoviridae family and is the most common cause of diarrhea observed in children in the 0–24-month age group.^3^ Oral vaccine applications against RV only became available in 2006. Administered orally, vaccination should be completed before children are 8 months of age.^4^ These vaccines are aimed at stimulating mucosal immunity as well as systemic responses.^5^ These may induce protective immunity not only against various infectious agents such as RV but may also pave the way for the development of new prophylactic and therapeutic ways to control or alleviate the severity of autoimmune diseases in humans.^5^

Since CD is an autoimmune disease, this has raised the question as to whether or not it and other autoimmune diseases such as type 1 diabetes mellitus (T1DM) are associated with RV and if the vaccine has impacted their subsequent incidence. This review discusses the possible effects of orally administered RV vaccine on the risk of developing CD and other autoimmune diseases.

## ROTAVIRUS VACCINE

Rotavirus is the most common cause of diarrhea in children aged 0–24 months.^3^ Highly infectious, RV is a double-stranded RNA virus from the Reoviridae family with three infectious protein layers.^6^ Viewed by electron microscopy, the three layers resemble a wheel (*roto* in Latin), leading to its name—rotavirus.

Rotavirus infection symptoms usually begin to appear within the first two days after exposure. Initial symptoms are fever and vomiting, followed by watery diarrhea for 3 to 8 days. During this period patients experience severe fluid loss. Diagnosis of RV is confirmed based on the patient’s clinical symptoms and physical examination.

Development of a vaccine was considered important since RV is the most common cause of gastroenteritis in the first two years of life and is highly contagious. The first marketable RV vaccine, administered orally, was released in 2006. To date, two RV vaccines have been licensed by the American Center for Disease Control and Prevention (CDC). RotaTeq® (RV5) (Merck, Madison, NJ, USA) is administered in three doses at 2, 4, and 6 months, and is a pentavalent bovine–human reassortant RV vaccine supplied in a 2 mL vial. Rotarix® (RV1) (GSK Biologics, Rixensart, Belgium) is administered in two doses at 2 and 4 months and is a monovalent human G1P RV vaccine supplied in a 1.5 mL vial.^7^ Immunity induced by either vaccine protects against homotypic and heterotypic RV strains.^8^ For either vaccine, the first dose should be given before the child is 15 weeks old; all subsequent doses should be completed before the child is 8 months old.^4^

These two vaccines have been licensed in more than 100 countries since 2006.^7^ Since 2017, RV vaccine has been included in the national routine infant vaccination programs of more than 80 countries.^7^,^9^

In recent years a few other RV vaccines have been developed. These include Rotasiil (Serum Institute of India Pvt. Ltd., Poon, India), a three-dose, pentavalent bovine–human reassortant vaccine that was nationally licensed in 2017, and ROTAVAC (Bharat Biotech International Ltd, Hyderabad, India), a three-dose, monovalent human–bovine G9P vaccine, licensed in 2014.^10^,^11^ Both of these were prequalified by the World Health Organization (WHO) in 2018.^12^ Two other live attenuated oral RV vaccines have been developed but are only licensed for domestic use in China (Lanzhou Lamb rotavirus vaccine; Lanzhou Institute of Biological Products, Lanzhou, China) and Vietnam (Rotavin-M1 rotavirus vaccine; Center for Research and Production of Vaccines and Biologicals Production Facilities, Hanoi, Vietnam).^11^

Vaccine efficacy and post-license efficacy studies conducted in high and upper-middle income countries have shown RotaTeq and Rotarix to be >90% effective in preventing severe RV disease.^13^–^18^ However, RV vaccine efficacy varied between 50% and 64% in studies conducted in countries with high infant mortality due to RV infection.^19^–^21^ The effectiveness of ROTAVAC in reducing hospitalization rates in India was 54%.^10^ The effectiveness of Rotasiil against hospitalization was 67% in Niger and 39% in India.^22^,^23^ The reason for the difference in vaccine efficacy between countries is not clear.

## EFFECT OF ROTAVIRUS VACCINE ON THE DEVELOPMENT OF CELIAC DISEASE

Oral vaccines offer a pain-free approach for inoculation to develop an appropriate immune response against microbial and other environmental antigens, either at the mucosal level or systemically.^5^ This approach, based on dietary antigens, can improve the systemic tolerance of the mucosal immune system against other autoantigens associated with environmental, dietary, and allergic autoimmune disorders.^5^ Furthermore, protective immune responses against various infectious agents can be induced by mucosal immunity. This may provide new prophylactic and therapeutic ways of controlling or alleviating the severity of autoimmune diseases in humans.^5^

Zanoni et al. have shown the RV infection to be a potential trigger for the onset of CD.^24^ They demonstrated that a subset of anti-TG IgA antibodies recognize a RV viral protein, VP7, and such antibodies increase small intestine permeability and induce monocyte activation.^24^ However, a cohort study conducted in the USA found that frequent clinically asymptomatic RV infections in children with human leukocyte antigen (HLA) risk alleles for CD lead to a higher risk for CD autoimmunity.^25^ Viruses other than RV have been implicated in the pathogenesis of CD. In an experimental study in mice by Bouziat et al., human reovirus infection triggered inflammatory responses to dietary antigens.^26^ In a different study, the same researchers found that CD patients tended to have a higher rate of anti-reovirus antibodies than the control group, but no significant difference in RV antibody levels was found.^26^

Kemppainen et al. found that CD autoimmunity increased within 3 months following gastrointestinal infectious episodes, especially in babies who ingested gluten in the first 6 months.^27^ In addition, RV vaccine administration reduced the risk of developing CD autoimmunity.

An important Finnish study was performed when the country introduced RV vaccine (RotaTeq) in their national immunization program.^28^ A total of 121,650 children (94,437 RV vaccinated, 27,213 RV unvaccinated) were included in the study. They identified 293 patients with CD, 201 of which were vaccinated. The study’s adjusted relative risk for CD was 0.89, but this was not significant. Hence, the researchers concluded that RV vaccine did not reduce the risk of developing CD in the first 4–6 years after vaccination. However, a limitation of this study was that follow-up was relatively short, the incidence of CD under 5 years of age is low, and the time needed to develop an autoimmune disease from induction may take several years.

A similar cohort study was conducted by Inns et al. in the UK and included 880,629 children (48.8% female).^29^ Rotarix was administered to 343,113 children, who were followed for an average of 5.1 years. The study identified 1,657 CD patients (incidence, 36.6 per 100,000 people). The adjusted hazard ratio for developing CD was 1.03 in vaccinated children. Girls had a 40% higher risk than boys. This and the study of Vaarala et al.^28^ have important similarities and differences: mean follow-up for both studies was 4–6 years, each study evaluated a different vaccine, and no significant risk for developing CD was detected. Clearly, both studies should be repeated with a longer post-vaccination period.

In 2015, Hemming-Harlo et al. conducted a long-term follow-up study of the Rotavirus Efficacy and Safety Trial.^30^ The original study, conducted between 2001 and 2003, evaluated RotaTeq versus placebo in more than 23,000 children 11–14 years after RV vaccination. Hemming-Harlo et al. sent a questionnaire to the parents of 19,133 participants, of which 30% responded. The survey revealed the development of CD in 48 (0.8%) of the participants. Noteworthily, the incidence of CD was higher in the placebo group versus vaccinated participants (1.1% versus 0.6%, respectively; *P*=0.027). In addition, the mean age at CD diagnosis was 5.3 years in the placebo group and 7.2 years in the RV vaccine group. The researchers concluded that the risk of CD autoimmunity decreased in children vaccinated with the RV vaccine. However, it is not known if this effect is permanent.

The pathogenesis of CD is multifactorial. Future studies should concentrate on different populations, countries, and RV vaccine types to more accurately evaluate the effect of vaccination on CD.

## OTHER AUTOIMMUNE DISEASES

### Type 1 Diabetes Mellitus

Type 1 diabetes mellitus is a chronic autoimmune disease characterized by insulin deficiency and hyperglycemia.^31^,^32^ It was thought to be a disease especially observed in children and adolescents, but recent studies have shown that the age of symptomatic onset is not a restricting factor for T1DM.^33^ The classic initial symptoms of T1DM are polydipsia, polyphagia, and polyuria.^32^ Anamnesis (classic initial symptoms: polyuria, polydipsia, polyphagia), physical examination, and laboratory tests (blood glucose levels, HbA1c) are used in the diagnosis. Exogenous insulin is mainly used in its treatment.

### Effect of Rotavirus Vaccine on the Development of Type 1 Diabetes Mellitus

The possible association between RV infection and the onset of T1DM was investigated by Honeyman and co-workers.^34^,^35^ Specifically, they evaluated the similarities between tyrosine phosphatase-like antigen 2 (IA-2) and glutamic acid decarboxylase 65 (GAD65) and the RV viral proteins. Both IA-2 and GAD65 are pancreatic islet autoantigens thought to be involved in T1DM pathogenesis. Honeyman et al. discovered significant similarities between epitopes within IA-2 and GAD65, and amino acid sequences of the RV viral protein 7 (VP7),^34^ which is known to be highly immunogenic. This suggests that antibodies created against VP7 in RV-infected individuals may have a role in the development of T1DM. In a subsequent study, they found that T cells had a proliferative response to RV and autoantigen-derived peptides.^35^ Their research pointed to molecular mimicry of T cell epitopes in rotavirus that could lead to the development of autoimmune diseases such as T1DM. Their ideas led to additional studies on the effects of the RV vaccine, for the purpose of preventing RV infection and protecting children from developing T1DM.^28^,^30^

Building on their earlier research, Honeyman et al. found that RV infection may indeed trigger T1DM and stressed the need to develop safe vaccines to protect against infection and development of this specific autoimmune response.^36^

This situation highlights the importance of taking precautions not only against RV infection but its consequences, such as development of T1DM. One example relates to Finland, which has the highest incidence of pediatric T1DM in the world.^37^,^38^ In order to evaluate the effectiveness of RV vaccination to also prevent pediatric T1DM, the same study by Vaarala et al. discussed above with regard to CD also looked at the incidence of T1DM among vaccinated and non-vaccinated children born in Finland between 2009 and 2010.^28^ The adjusted relative risk for T1DM was found to be 0.91, and researchers found no difference in the risk for T1DM within the first 4–6 years after oral RV vaccination (RotaTeq).^28^ The same limitations apply as discussed above and clearly demonstrate the need to investigate more types of RV vaccination and perform long-term follow-up to determine the true effect of RV vaccination in preventing development of T1DM.

The 2015 Finnish study by Hemming-Harlo et al. discussed above also evaluated the risk of developing T1DM 11–14 years after RV vaccination.^30^ The study revealed the development of T1DM in 58 respondents and of type 2 DM in 1 respondent. Development of T1DM was 0.97% for the placebo group and 1.04% in the RotaTeq vaccine group.^30^ However, no significant difference was found between the two groups (*P*=0.810), although the authors noted that during the follow-up period T1DM incidence increased steadily in both vaccinated and unvaccinated children.

The Australian study by Perrett et al. compared the period before and after RV vaccination.^39^ In contrast to the Vaarala et al. study in Finland,^28^ Perrett et al. noted a 15% decrease in the incidence of T1DM in the 0–4 age group after vaccination,^39^ with no significant difference in the incidence in older age groups. Although the authors do not mention the type of RV vaccine, an Australian governmental website^40^ indicates that RotaTeq was used from 2007 until 2017; hence, only one vaccine type was examined in the study by Perrett et al.

A 2019 study in the USA included both RotaTeq and Rotarix^41^ and noted a 33% decrease in the incidence of T1DM in fully vaccinated children versus unvaccinated children. Furthermore, RotaTeq was found to have a stronger effect than Rotarix. However, a different study, also in the USA but in 2020, found no difference in T1DM incidence in vaccinated versus unvaccinated children that were followed from 1 to 12 years of age after RV vaccination.^42^ Differences between these two studies could be due to inclusion criteria, environmental differences, dietary habits, sociocultural differences, population differences, and genetic variations.

Future studies on the effect of RV vaccine on T1DM development risk should include data such as children’s body structures, body mass index, sociocultural structures, and dietary habits. In addition, in order to determine the causes of differences between populations, the dominant factors in the pathogenesis of T1DM should be examined within the studied populations. Only then can it be determined if and how the RV vaccine affects T1DM pathogenesis.

### Rheumatoid Arthritis, Thyroiditis, and Inflammatory Bowel Diseases (Crohn’s Disease and Ulcerative Colitis)

Of the above studies, only Hemming-Harlo et al. evaluated possible associations between the RV infection and vaccination, and autoimmune diseases other than CD and T1DM.^30^ Rheumatoid arthritis was detected in 30 of 5,764 subjects (19/3,184, RV vaccine group; 11/2,580, placebo group); there was no difference in disease prevalence between the two groups. The number of patients found in their study with thyroiditis, Crohn’s disease, and ulcerative colitis was very low, raising the question of whether RV infection or vaccination has any role in these diseases.

## CONCLUSION

This review has examined the effects of RV vaccine on the risk of developing CD and other autoimmune diseases. Table 1 summarizes the studies examined in this review. The studies discussed herein suggest that the risk of developing CD may be lower in individuals who have received the RotaTeq RV vaccine, and that presentation of the disease was delayed. Despite conflicting evidence on the effect of RV vaccine on the risk of developing T1DM, RV vaccination may—at the least—delay its onset. In general, there currently seems to be no indication that RV or RV vaccination has an effect on other autoimmune diseases, but this issue has not been adequately explored.

**Table 1:** Summary of Key Findings of Studies Examined in this Review.

Study	Country	Post-vaccination Risk for:			Post-vaccination Follow-up (y)
		CD	T1DM	Other	
2017^28^	Finland	IRR 0.87 (CI 0.65–1.17)	IRR 0.91 (CI 0.69–1.20)	Not examined	4–6
2019^30^	Finland	Prevalence 0.60% (CI 0.38–0.93) (Placebo group 1.11%)	Prevalence 1.04% (Placebo group 0.97%)	No difference for rheumatoid arthritis*	11–14
2019^39^	Australia	NS	IRR 0.85 (CI 0.75–0.97)	Not examined	0–4
2019^41^	USA	NS	HR 0.67 (CI 0.54–0.83)	Not examined	0–5
2020^42^	USA	NS	HR 1.09 (CI 0.87–1.36)	Not examined	1–12
2021^29^	UK	HR 1.05 (CI 0.86–1.28)	HR 0.89 (CI 0.68–1.19)	Not examined	0–5

* The study did not have enough patients to examine thyroiditis and inflammatory bowel disease.

CD, celiac disease; CI, confidence intervals; HR, hazard ratio; IRR, incidence rate ratio; NS, not statistically significant; T1DM, type 1 diabetes mellitus; UK, United Kingdom; USA, United States of America.

Many questions remain unanswered with regard to the effect of RV disease and vaccination, such as the duration of the protective effect, differences between the various RV vaccinations, and whether or not other demographic, socioeconomic, and other personal confounding variables have a role in the development of autoimmune conditions. Future study designs should take these factors into consideration. Additional studies, however, are not enough. There needs to be a concerted and unified international effort to investigate the most commonly administered RV vaccines to definitively answer these important questions.

## Abbreviations

CD: celiac disease
CDC: Centers for Disease Control and Prevention
GAD65: glutamic acid decarboxylase 65
HLA: human leukocyte antigen
IA-2: tyrosine phosphatase-like antigen 2
RV: rotavirus
T1DM: type 1 diabetes mellitus
USA: United States of America
VP7: viral protein 7
